# Novel artificial intelligence assisted Landsat-8 imagery analysis for mango orchard detection and area mapping

**DOI:** 10.1371/journal.pone.0304450

**Published:** 2024-06-14

**Authors:** Muhammad Usama Tanveer, Kashif Munir, Ali Raza, Mubarak S. Almutairi

**Affiliations:** 1 Institute of Information Technology, Khwaja Fareed University of Engineering and Information Technology, Rahim Yar Khan, Pakistan; 2 Department of Software Engineering, University of Lahore, Lahore, Pakistan; 3 College of Computer Science and Engineering, University of Hafr Al Batin, Hafr Alabtin, Saudi Arabia; AUM: American University of the Middle East, KUWAIT

## Abstract

The mango fruit plays a crucial role in providing essential nutrients to the human body and Pakistani mangoes are highly coveted worldwide. The escalating demand for agricultural products necessitates enhanced methods for monitoring and managing agricultural resources. Traditional field surveys are labour-intensive and time-consuming whereas remote sensing offers a comprehensive and efficient alternative. The field of remote sensing has witnessed substantial growth over time with satellite technology proving instrumental in monitoring crops on a large scale throughout their growth stages. In this study, we utilize novel data collected from a mango farm employing Landsat-8 satellite imagery and machine learning to detect mango orchards. We collected a total of 2,150 mango tree samples from a farm over six months in the province of Punjab, Pakistan. Then, we analyzed each sample using seven multispectral bands. The Landsat-8 framework provides high-resolution land surface imagery for detecting mango orchards. This research relies on independent data, offering an advantage for training more advanced machine learning models and yielding reliable findings with high accuracy. Our proposed optimized CART approach outperformed existing methods, achieving a remarkable 99% accuracy score while the k-Fold validation score also reached 99%. This research paves the way for advancements in agricultural remote sensing, offering potential benefits for crop management yield estimation and the broader field of precision agriculture.

## Introduction

Mango ranks second on the list of important fruits produced in Pakistan in terms of area and productivity. According to the Ministry of Commerce and Trade, Pakistan produces 1.75 million tons of mangoes each year [1]. In the past, the country was able to export over 100,000 tons of mangoes during the 2005–2006 and 2012–2013 periods, accounting for around six percent of its production. The provinces of Sindh and Punjab are the main exporters of mango fruit [2]. With the population growing daily proper food management has become imperative. Pakistan’s agricultural sector is currently striving to increase mango yield [3]. However, the sector faces challenges in producing reliable data, and our work aims to address the data gap on mango orchards using contemporary techniques. A higher degree of proficiency is required to implement varietal modification [4].

The agriculture industry is expanding and changing daily in the modern era [5]. The majority of research farms are transitioning from traditional agricultural methods to smart solutions. Since 2001, China’s Agriculture Remote Sensing Monitoring System (CHARMS) has been monitoring crops [6]. They monitor soil moisture, vegetation, assess humidity and predict agricultural yields using remote sensing devices. They also incorporate weather data. This method aids in generating insightful outcomes. Landsat 8’s multi-spectral bands CHARMS employs a hierarchical classification strategy tailored specifically to the distinctive spectral characteristics of mango trees. By integrating various image processing techniques such as feature extraction spectral signature analysis and machine learning algorithms, CHARMS can accurately differentiate mango trees from surrounding land cover types with high precision. This approach is particularly advantageous in regions where mango cultivation is prevalent providing valuable insights into orchard distribution health assessment and yield estimation. CHARMS offers scalability and adaptability allowing for its potential application in other fruit tree classification tasks beyond mangoes. With its robust methodology and reliable results CHARMS stands as a promising tool for agricultural monitoring and management, facilitating informed decision-making and sustainable practices in the mango cultivation sector.

One distinctive application of Landsat 8 satellite imagery [7] compared to Sentinel satellites lies in its utilization for precision agriculture, particularly in regions with smaller landholdings or fragmented landscapes. Landsat 8’s moderate spatial resolution (30 meters) and spectral bands enable detailed monitoring of crop health, soil moisture content, and vegetation vigor at a finer scale. This capability is particularly beneficial for smallholder farmers and agricultural cooperatives who may lack access to high-resolution imagery or costly precision agriculture technologies. By integrating Landsat 8 data with ground-based information and agronomic models, farmers can make informed decisions regarding irrigation scheduling, fertilizer application, and pest management, leading to optimized yields and sustainable land use practices. Additionally, Landsat 8’s long-term archive facilitates historical analysis and trend identification, aiding in the assessment of agricultural productivity changes over time.

Machine learning (ML) models such as Support Vector Machines (SVM), Random Forests (RF), Naive Bayes (NB) Classification and Regression Trees (CART) and k-Nearest Neighbors (kNN) offer significant benefits for mango tree detection in classification tasks [8]. SVM excels in handling high-dimensional data and can effectively separate mango trees from other land cover types by finding an optimal hyperplane. RF on the other hand, utilizes an ensemble of decision trees to improve accuracy and generalization making it robust against noise and overfitting. NB, with its simple probabilistic framework, is computationally efficient and suitable for large datasets, making it valuable for mango tree detection in resource-constrained environments. CART, known for its interpretability, generates decision trees that can easily be understood and visualized, aiding in the identification of key features for mango tree classification. Additionally, kNN relies on proximity-based learning, making it suitable for spatially clustered mango orchards where neighbouring trees exhibit similar spectral signatures. By leveraging these ML models, mango tree detection tasks benefit from enhanced accuracy, efficiency, and scalability, facilitating precise mapping and monitoring of mango orchards for agricultural management and decision support purposes.

Our innovative contribution to this study is as follows:

- We adopt a novel approach to data collection using the Landsat-8 satellite from the desired location. The collected dataset is utilized to detect mango trees, perform classification based on Landsat-8 satellite data, and map the areas accordingly. This methodology allows for precise identification and categorization of mango orchards.
- We deployed five advanced machine learning models and trained them to achieve higher performance accuracy. We also performed state-of-the-art comparisons and applied the K-Fold Validation technique to validate our data gathered from satellites. We used a hyperparameter tuning approach for the applied machine learning models to enhance performance.

The scientific inquiry is divided into portions as follows: Section “Literature Analysis” analyses existing literature on mango tree Classification. Section “Proposed methodology” evaluates our study approach. Section “Results and discussions” focuses on the scientific validation and evaluation of our study methodology. Section “Conclusion and future work” summarises the research findings.

## Literature analysis

This section examines a variety of tactics and methodologies for anticipating in the literature and a summary is provided in Table 1.

**Table 1:** The literature analysis based on state-of-the-art approaches performance.

Ref	Year	Satellite	Proposed Technique	Performance Score
[9]	2019	Landsat-8 and Sentinel	MLP	95%
[7]	2020	Landsat-8, OLI	Pricipal component Analysis	92%
[10]	2014	Landsat-8, ETM	CDC	90%
[11]	2020	Landsat-8	Support vector Machine	92%
[12]	2023	Landsat-8	Convolutional Neural Network	84%
[13]	2018	Landsat-7	Computer viison	92%
[14]	2022	Landsat-8	Pricipal component Analysis	95%
[15]	2022	Sentinel-1 and 2	Random Forest	91%
[16]	2022	Sentinel-2	Support vector machine	91%
[17]	2022	Sentinel 1 and 2	Random forest	93%
[18]	2022	sentinel 1	Decision Tree	90%
[19]	2019	Landsat-7	Random forest	91%

Sentinel-2 data is more effective for analyzing forest [9] characteristics than Landsat 8 imagery, resulting in lower root mean square error values and higher coefficients of determination (R^2^). Interestingly, there was no discernible difference in systematic error between the two instruments. The test dataset shows consistent minimum systematic error across all forest variable models and satellite datasets. The Multilayer Perceptron (MLP) modelling method produced little bias across most models. Surprisingly, the addition of Landsat 8 image bands to Sentinel-2 data did not enhance prediction accuracy over models that only used Sentinel-2 bands. The use of all image bands or only the 20-m resolution bands of Sentinel-2 resulted in nearly equivalent prediction accuracies, demonstrating Sentinel-2’s robustness in forest variable modelling scenarios.

Remote sensing presents challenges in detecting [10] land cover change and classification, necessitating improved methods for leveraging the temporal domain of Landsat data to enhance accuracy in both tasks. This study addresses these challenges by introducing a novel algorithm tailored for continuous change detection and classification at high temporal frequencies. Through the utilization of all accessible Landsat data, this approach enables the reconstruction of Earth’s surface history spanning the Landsat TM and ETM+ era. Notably, the models are estimated using sine functions, enabling a comprehensive understanding of temporal patterns and changes in land cover over time. This innovative methodology offers promising prospects for advancing the precision and comprehensiveness of land cover change detection and classification efforts in remote sensing applications.

A novel methodology has been devised to augment [7] the accuracy of change detection within mango fruit crop areas utilizing bi-temporal Soil Adjusted Vegetation Index (SAVI) images. This approach encompasses a two-step procedure: firstly, the derivation of log-ratio (LR) and principal component (PC) images from SAVI data captured in 2015 and 2019, respectively, followed by the merging of these images on a pixel-by-pixel basis; secondly, the classification of the fused images into ‘positive change,’ ‘no change,’ and ‘negative change’ categories using a predefined threshold value. The outcomes reveal a significant enhancement in change detection accuracy, with the LR-PCA method achieving an impressive 92% accuracy rate. Comparative evaluation against conventional change detection techniques including vegetation image differencing, image rationing, PCA and log-ratio reaffirms the superiority of the proposed LR-PCA approach.

In pursuit of heightened classification accuracy within [11] the Support Vector Machine (SVM) framework an examination of three distinct kernel functions was undertaken: Radial Basis Function, Sigmoid kernel and Polynomial kernels (Udgata et al., 2020). The investigation revealed that SVMRBF emerged as the most efficacious classification approach, boasting an impressive overall accuracy of 92.44% and a kappa coefficient of 0.9218. Notably, employing SVMRBF for the classification of satellite imagery successfully delineated mango plantations. This study underscores the significance of leveraging satellite data alongside advanced classification algorithms for precise computation of mango acreage, with SVMRBF standing out as the foremost method in terms of effectiveness.

This study unambiguously illustrates [12] the effectiveness of Machine Learning (ML) algorithms in rapidly and automatically estimating commercially crucial horticulture crops like mango and coconut. The innovative techniques used to extract information from remotely sensed photos are critical to the development of intelligent horticulture information systems. These technologies in turn help to make educated decisions about the export/import dynamics of these commodities, improving the overall efficiency of their supply chain. The Convolutional Neural Network (CNN) model performed particularly well, achieving an accuracy rate of 84%. This accomplishment in precisely mapping coconut production demonstrates the potential of machine learning algorithms to change crop estimating methods, allowing for more prompt decision-making and resource management in the horticulture sector.

The Hyperion hyperspectral satellite data was thoroughly analyzed to identify the extent of mango orchards in the area [13]. A comparative study with ground-truth data provided from GPS measurements demonstrated a good level of accuracy with the anticipated total mango orchard size of 961.88 hectares. This estimate was 92% accurate using the actual ground data of 889.65 hectares. The findings highlight the potential and usefulness of hyperspectral remote sensing as a helpful tool for accurately estimating fruit crop acreage, demonstrating its importance in modern agricultural practices and decision-making processes.

The evaluation focused on four major change detection methods: Vegetation Index Differencing (VID), Log Ratio, Principal Component Analysis (PCA), and Image Ratioing (IR), with the goal of identifying changes in the mango crop area [14]. To designate the mango zones, Soil Adjusted Vegetation Index (SAVI) pictures from 2015 and 2019 were generated and then used to generate VID, LR, PCA and IR raster images. The precision of the change detection systems was carefully tested, and the overall accuracy was found to be an amazing 95%. This study provides useful insights into the ideal change detection methods for mango fruit crops, hence improving the efficacy of monitoring and management measures in agricultural landscapes.

In mango orchard recognition [15], Random Forest (RF) outperformed Support Vector Machine (SVM); however, the differences were not statistically significant. As a result, SVM is still a feasible alternative for Land Use and Land Cover (LULC) classification, particularly when using robust variable selection methods like relief. Given the conflicting results of previous studies on classifier and variable selection performance, an ensemble approach is advocated. This strategy takes advantage of the capabilities of each classifier and predictor variable set, resulting in a more balanced and accurate classification process. The study’s overall accuracy score was an astounding 90%, highlighting the potential of integrated approaches in improving mango orchard mapping and classification.

Combining Synthetic Aperture Radar [16] (SAR) data (S1) and optical data (S2) with machine learning classifiers appears to be an effective method for mapping fruit-tree crops in subtropical areas with frequent cloud cover. This study shows that the Support Vector Machine (SVM) classifier achieves the best classification results for both fruit-tree crops and co-existing land uses. The SVM classifier delineates various land cover classes with improved accuracy by utilising six essential factors, one of which is from the S1 channel (VH polarisation) and scores 91.

This study fills that gap by focusing on the integration of optical [17] and (SAR) data into a categorization framework. The study methodically determines the best time frame and input layers for the Random Forest (RF) classifier resulting in an amazing 93% accuracy rate. This integrated technique not only improves the precision of land cover mapping but also demonstrates the possibility of combining several data sources to improve classification outcomes, particularly for tree-fruit crop types.

The findings of this study demonstrate the feasibility [18] of attaining more precise discrimination among varieties of soybeans by using spectral bands from publically available photos, such as those acquired by the Landsat-8 satellite, as input for evaluated machine learning models. This advancement in soybean mapping is a big step toward accurately identifying the main cultivars within a given region. Nonetheless, in order to improve the efficacy of plant species recognition, more orbital data should be explored, with a focus on cloud-free observations. This can be accomplished using either enhanced spatial resolution datasets, such as Sentinel-2/MSI, or satellite constellations, paving the path for future advances in soybean genotype mapping.

The original cluster has a more diversified time series [19] with changes in phenology at each stage of the fruiting cycle and increased NDVI values, possibly due to the development of new leaves following pruning. The measurement of NDVI values over time for mango trees thus emerges as a viable tool for distinguishing between trees experiencing active or dormant phenological changes within a given time frame. The possible development of this work could help monitor the state of mango trees and plantations, providing significant insights to the Department of Agriculture. Such insights may be useful in the rehabilitation of mango trees in the province, fostering a healthier and more sustainable mango business.

### Research limitations

Studies that are now available on mango tree detection have several shortcomings Which can be summed up like this:

- Previous studies do not discuss Data acquisition techniques from Satellite.
- Previous researchers used one or two ML classical models. However, using five advanced machine learning models will increase the accuracy of the Findings.
- Previous studies Accuracy scores range from 80 to 95 per cent, suggesting that more work has to be done to reach the best possible outcomes.
- Past researchers did not evaluate the Hyperparameters of Deployed models. We evaluate ML models against one another.

## Proposed methodology

**Fig 1:**
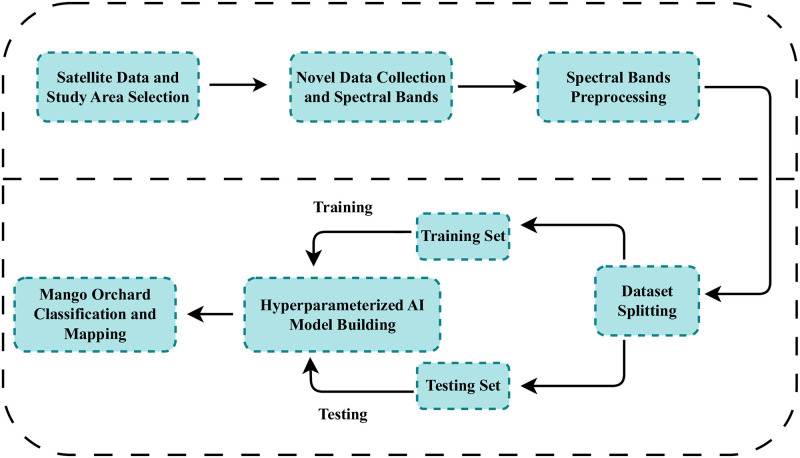
Our study purposed methodology.

The workflow of our study is illustrated in Fig 1. Our study utilizes satellite data and feature datasets to train machine-learning models. Feature engineering involves utilizing satellite band data and conducting Exploratory Data Analysis to identify patterns and key factors for information extraction. During the feature engineering phase the dataset undergoes band preprocessing. Following preprocessing, the dataset is split into two parts: the training set and the testing set, with an 80/20 split ratio. Various advanced machine learning and deep learning models are compared. After parameterization, the best-performing model undergoes rigorous training and testing. Subsequently, the proposed model is employed for detecting mango orchards using the Landsat 8 image dataset.

### Phase 1: Selection of study area

**Fig 2:**
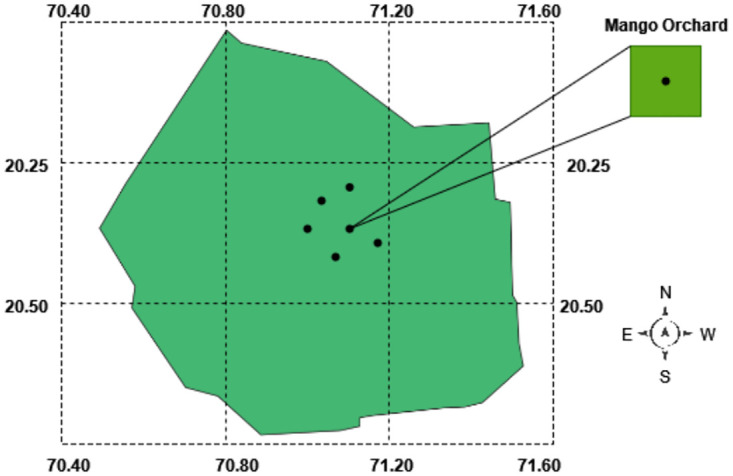
Our study location coordinates 28.25278 N, 70.09366 E.

We have discovered our cutting-edge research facility nestled in the picturesque Mango Farm, situated in the heart of the district in the province of Punjab, as shown in Fig 2. Renowned for its prolific production of succulent mango fruits and the meticulous cultivation of mango trees, this farm serves as a beacon of innovation. Our study area contains various types of mango trees and the surrounding area is covered with different crops such as wheat, cotton and rice. This mango farm contains 725 different species of mango trees. Two hundred trees are less than one year old, four hundred plants are more than four years old and the remaining plants are more than three years old. The weather conditions in this city are notably hot in summer. The Mango Farm is an ideal subject for this exploration, boasting representatives from all Land Use Strategy classes. This unique attribute empowers us to conduct a meticulous analysis unraveling the nuances of policy mappings with unmatched precision.

### Phase 2: Novel data collection and spectral bands

The data collection is subjected to a technique known as orthorectification, which corrects distortions caused by topographical differences to provide accurate spatial representation. A Digital Elevation Model (DEM) is used to adjust for topography variations. After geometric rectification, Landsat 8 imagery can be expressed in geographic coordinates, commonly represented as latitude and longitude. In addition, the pixel values are resampled to meet the specified spatial resolution. Geographic Information System (GIS) software [20] is often used for these tasks, allowing users to interact with georeferenced data. In this process, we convert Landsat 8 bands into data coordinates that can perform accurate spatial analysis overlay datasets and integrate satellite imagery with other geospatial information for various applications such as land cover mapping, environmental monitoring and natural resource management.

We used Landsat 8, which provides high-resolution imagery, with most bands having a spatial resolution of 30 metres [21]. This degree of detail is useful for tracking changes in land cover, detecting environmental trends and supporting a variety of applications such as agriculture forestry and urban planning [22]. Landsat8 data is freely available to the public via the USGS Earth Explorer website. This open data policy encourages extensive use of satellite data for research monitoring and making decisions [23].

Then, Landsat-8 bands are converted into data coordinates [24] using a procedure known as a geometric correction, which matches the satellite imagery with real-world geographic coordinates. Landsat-8 data is first obtained in raw pixel coordinates, and converting it to geographic coordinates requires multiple stages. The first stage uses sensor-specific geometric adjustments to compensate for distortions caused during picture acquisition. This adjustment method considers the position of the spacecraft and sensors as well as the rotation of the Earth.

**Fig 3:**
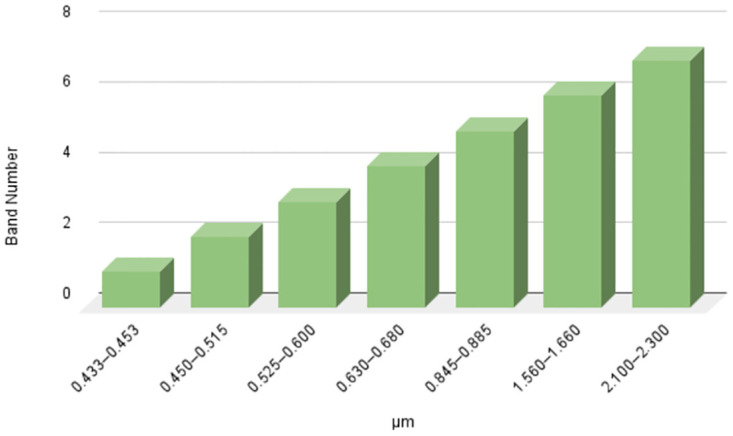
Our purposed study shows statical analysis of Lansat8 bands.

Fig 3 Shows the lansat8 Band Statical Analysis. The Operational Land Imager on Landsat 8 is equipped with seven distinct spectral bands, each designed to capture specific details about the Earth’s surface [25]. Band 1, known as the Coastal Aerosol band, spans the ultra-blue spectrum and focuses on the properties of shallow water and aerosols. Band 2, the Blue band, offers insights into water quality and penetration while Band 3, the Green band, is particularly sensitive to changes in vegetation health and land cover [26]. Emphasizing the vitality of vegetation, especially in agricultural contexts, is Band 4, also known as the Red band. Band 5, the Near Infrared (NIR) band, plays a crucial role in assessing vegetation moisture content, contributing to a comprehensive analysis of vegetation [27]. Bands 6 and 7, Shortwave Infrared 1 and Shortwave Infrared 2, respectively, delve into studies related to geology and vegetation, with SWIR 2 having the capability to penetrate through atmospheric conditions [28]. Collectively, these seven bands provide a diverse dataset enabling applications such as land cover classification, vegetation monitoring and the detection of environmental changes. Researchers utilize this wealth of spectral information to unravel intricate details about the Earth’s surface and to monitor dynamic processes unfolding over time.

The novel collection contains data from seven separate bands [29] organized in a CSV format with 2150 rows. Each row represents a single observation or instance, while the columns relate to various aspects or variables inside each observation. This tabular structure allows for the orderly organization of data, which aids in analysis and interpretation. The seven bands offer a multidimensional picture capturing differences across spectral ranges or characteristics. This dataset’s structured architecture lends itself to various analytical tools, making it a significant resource for researchers, analysts, and scientists looking for insights into remote sensing environmental monitoring and data-driven decision-making.

### Phase 3: Bands data spitting

To prevent applied model overfitting and to evaluate the trained model on an unobserved test segment of the dataset, the data is split using the data splitting method. The dataset, including mango trees, has been cut in half for training and testing machine learning models. The ratio of 80:20 is utilized for partitioning the dataset. A component of the dataset 80% is used for the training of the model. In contrast, another portion 20% of the dataset, is utilised to evaluate the used model’s performance on data that has not yet been seen. Our research models have been trained and tested, and the findings have shown high accuracy.

### Phase 4: Artificial intelligence techniques

Machine learning techniques are pivotal in enhancing Artificial Intelligence (AI)-assisted Landsat-8 image analysis for detecting and mapping mango orchards. By leveraging sophisticated algorithms, such as Rf, KNN, NB, Svm and Cart these techniques can accurately interpret the complex spectral signatures unique to mango orchards from satellite imagery.

- **Support vector Machine (SVM)**: In agricultural management [30] the application of Support Vector Machines (SVM) in detecting mango trees using satellite data has emerged as a significant advancement. SVM a supervised learning algorithm proves effective in identifying mango trees amidst diverse features present in satellite imagery. By training the SVM model with labelled data representing mango tree features and other elements such as soil or vegetation, the algorithm learns to classify pixels accurately. The decision boundary established by SVM maximizes the margin between different classes, enhancing the model’s precision. The SVM equation, which governs its functioning, is expressed as:
  $$\begin{array}{c} \underset{w,b}{\text{min}}\frac{1}{2}{∥w∥}^{2}+C\sum_{i=1}^{n}\;\text{max}\left(0,1-y_{i}\left(w\cdot x_{i}-b\right)\right) \end{array}$$ (1)
  Here, w denotes the weights, b represents the bias term, C is the regularization parameter, xi signifies the input features, and yi indicates the class label. This equation enables the SVM algorithm to effectively distinguish mango trees from other elements in satellite data thereby facilitating efficient agricultural monitoring and management practices.
- **Random Forest (RF)**: In agricultural management the application of Random Forest models [16] for mango tree detection using satellite data has emerged as a valuable tool. Random Forest, an ensemble learning technique, is particularly adept at analyzing complex datasets such as satellite imagery. By leveraging multiple decision trees trained on various features Random Forest can effectively discern mango trees from other elements present in the imagery. Through the process of ensemble learning, where predictions from multiple models are aggregated Random Forest enhances its accuracy and robustness. Although Random Forest does not have a single equation like some other algorithms its underlying principles involve the construction and combination of decision trees to make predictions. This approach contributes significantly to agricultural monitoring and management providing farmers with valuable insights to optimize their resources and improve crop yield. The mathematical equation as follows:
  $$\begin{array}{c} \hat{y}=\text{mode}(f_{1}\left(x\right),f_{2}\left(x\right),\ldots ,f_{T}\left(x\right)) \end{array}$$ (2)
- **K-Nearest Neighbour (KNN)**: The utilization of the K-Nearest Neighbors (KNN) [30] algorithm for detecting mango trees using satellite data has emerged as a promising approach due to its simplicity and effectiveness. KNN a non-parametric and instance-based learning method classifies new data points based on the majority class of their k-nearest neighbors in the feature space. In the context of mango tree detection KNN starts by extracting relevant features from satellite imagery, such as spectral bands and texture information. Subsequently, for each pixel in the satellite image KNN computes the distance to its k-nearest neighbours in the feature space and assigns it to the class that is most prevalent among those neighbours. This process enables KNN to discern mango trees from other elements present in the satellite data. The mathematical equation governing the KNN algorithm is as follows:
  $$\begin{array}{c} \hat{y}=\text{mode}(y_{1},y_{2},\ldots ,y{)}_{k} \end{array}$$ (3)
  Here, y denotes the predicted class label for a given data point, and y1, y2, …,y3, y4, …, yk represent the class labels of its k-nearest neighbours. Leveraging the KNN model, agricultural stakeholders can efficiently monitor mango tree distribution and optimize management strategies to enhance crop yield and resource utilization.
- **CART**: The Classification and Regression Trees (CART) model [31] is widely applied to detect mango trees using satellite data due to its adaptability and interpretability. CART functions by recursively segmenting the feature space, making it particularly adept at handling the complexities inherent in satellite imagery analysis. When tasked with mango tree detection CART utilizes various spectral and spatial features extracted from satellite data to construct a decision tree. This tree comprises nodes representing feature thresholds and branches depicting binary decisions based on these thresholds. Let j be the index of the feature used for splitting at node m and s be the threshold value for the split. The CART model can be represented by a series of binary decision rules:
  $$\begin{array}{c} \begin{array}{cc} & \text{If}\;x_{j}\leq s,\;\text{then}\;\text{left}\;\text{child}\;\text{node}\\ & \text{If}\;x_{j}>s,\;\text{then}\;\text{right}\;\text{child}\;\text{node} \end{array} \end{array}$$ (4)
  At each node, the decision is made based on whether the value of the selected feature xj is less than or equal to the threshold s. This process continues recursively until a stopping criterion is met maximum tree depth minimum samples per leaf node or others.
- **Naive Bayes (NB)**: The Naive Bayes model serves [32] as a prevalent method in detecting mango trees using satellite data owing to its straightforwardness and effectiveness. Founded on Bayes’ theorem this model calculates the probability of an event’s occurrence considering prior knowledge of conditions that might be pertinent to the event. In the context of mango tree detection, the Naive Bayes model operates under the assumption that features derived from satellite data are conditionally independent given the class label—whether mango trees are present or absent. This simplifying assumption enables the model to ascertain the probability of a pixel belonging to the mango tree class by assessing the likelihood of observing its features given the presence of mango trees, coupled with the prior probability of mango trees existing within the dataset. The Naive Bayes model can be expressed as:
  $$\begin{array}{c} P\left(y|x_{1},x_{2},\ldots ,x_{n}\right)=\frac{P\left(x_{1},x_{2},\ldots ,x_{n}\right)P\left(y\right)}{\prod_{i=1}^{n}P\left(x_{i}|y\right)} \end{array}$$ (5)

### Phase 5: Hyperparameter tuning

Table 2 Shows Our study’s proposed Hyperparameter Analysis. We intend to determine the optimal hyperparameters for each machine-learning method by continually training and testing them. Once determined, these hyperparameters enable the machine learning model to deliver reliable prediction results. The assessment of hyperparameter modifications for our study models is detailed in Table 2, which depicts the criteria used to get remarkable performance metric scores. The revised hyperparameters significantly benefit this research study’s Machines for Computing models.

**Table 2:** Our study shows hyperparameter values of applied models.

Method	Hyperparameter	Descriptions
*KNN*	‘n_*n*_*eighbour*=5, *weights*=′*uniform*′, *metric*=′*minkoski*′, *leafsize*=30, *p*=′ 2′	n_neighbors = 5 to consider the five nearest points in the voting process, and uses a uniform weighting scheme where each neighbor contributes equally. It employs the Minkowski metric with p = 2, equivalent to the Euclidean distance, and optimizes neighbor searches with a leaf_size of 30.
*SVM*	kernel=’linear’, C=’1.0’, degree=3, gamma,’scale’, tol=le-3, cache_*s*_*ize*=200, *decision* – *function* – *shape*=′*ovr*′.	kernel=’linear’ uses a linear kernel function, suitable for data that can be linearly separated, and is regulated by C = 1.0, which balances the trade-off between achieving a low error on the training data and minimizing model complexity.
*NB*	var_*s*_*moothing* = *le* − 9	var_smoothing parameter in Naive Bayes models is used to add a small value (1e-9) to the variance of the features to stabilize calculations and avoid division by zero errors.
*RF*	max_*d*_*epth*=20, *random*_*s*_*tate*=0, *n*_*e*_*stimators*=100, *criterion*=′*gini*′, *max*_*f*_*eatures*=′*sqrt*′.	maximum tree depth of 20, a random seed of 0, 100 trees, the Gini impurity criterion, and the maximum number of features considered at each split set to the square root of the total features.
*Cart*	max_depth=2, random_state=500, criterion=’entropy’	maximum tree depth of 2, a random state seed of 500, and uses entropy as the criterion for splitting nodes.

## Results and discussions

In this section, we will look at the results of our suggested research study and the scientific evaluations of those data. The accuracy score the precision score the recall score and the f1-score are the performance measures utilized. Our research models are being evaluated for their level of scientific plausibility based on the performance indicators.

### Experimental setup

The following constitute the primary elements of evaluation metrics:

- The correlation between the anticipated and actual values is referred to as a true positive (TP).
- Both the anticipated and actual values are negative, referred to as a true negative (TN).
- The term false positive refers to a situation in which the actual value is negative but the anticipated value is positive. (FP).
- In this case, the real value is positive, but the predicted value is negative; this is a false negative abbreviated as FN.

The accuracy score [33] of the model being utilised demonstrates the degree to which the model successfully predicts. The error rate of a model is also connected to the accuracy of the model. The more accurate the measurement, the lesser the percentage of mistakes. To calculate how accurate a forecast is, simply divide the number of correct predictions by the total number of guesses. The accuracy score of the model that we have proposed is a perfect 100. The accuracy score can be shown in a mathematical demonstration as:

$$\begin{array}{c} AccuracyScore=\frac{TP+TN}{TP+TN+FN} \end{array}$$ (6)

The positive predictive value of a learning model is another name for the precision score [34] of that model. The proportion of correctly predicted positive labels is used as a metric for determining the level of precision. The precision, in general, is a calculation that determines the accuracy of the model used to predict a data sample as positive. The proposed model that we developed has a precision score of 100 percent. The following is a list of the mathematical notations that can be used to express precision scores:

$$\begin{array}{c} PrecisionScore=\frac{TP}{TP+FP} \end{array}$$ (7)

The recall score [35] of employed models measures how many of the TP were recalled (found) properly. This score is expressed as a percentage. The recall of a learning model is sometimes referred to as the model’s sensitivity. The proposed model that we developed has a recall score of 100 percent. The recall can be denoted using the following mathematical notations:

$$\begin{array}{c} RecallScore=\frac{TP}{TP+FN} \end{array}$$ (8)

The f1 score [36] is a statistical measure that summarises the performance of a prediction model by combining the values of precision and recall. This score is derived from the F1 statistic. The f1 measure is the harmonic mean that is calculated by averaging the precision and the recall. Our proposed model has a perfect score of 100 percent on the f1 scale. The mathematical expression that represents the equation used to determine the f1 score is as follows:

$$\begin{array}{c} F1Score=2*\frac{\left(Precision*Recal\right)}{\left(Precison+Recal\right)} \end{array}$$ (9)

### Performance results of applied methods

**Fig 4:**
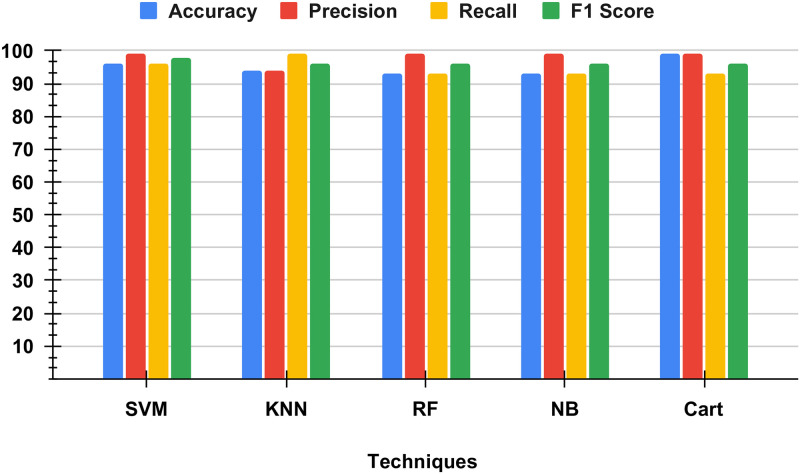
Our study shows accuracy, precision, recall, and F1 score.

Table 3 and Fig 4 Show Performance Analysis of Applied Methods. An investigation into the relative merits of various performance criteria for applied learning models. The results of the computations involving time complexity and performance metrics are calculated without using the approach that we have proposed. According to the study’s findings, all of the applied learning models reached average scores when predicting Target. The highest Accuracy precision and F1 score achieved by random forest and Decision tree model 99%. The minimum accuracy is 94% achieved by Naive Bayes; however, low-performance metrics Score.

**Table 3:** Shows time, accuracy, precision, F1 score and recall.

Technique	Accuracy	Precision	Recall	F1
*SVM*	96	99	96	98
*KNN*	94	94	99	96
*RF*	93	99	93	96
*NB*	93	99	93	96
**Cart**	99	99	93	96

**Fig 5:**
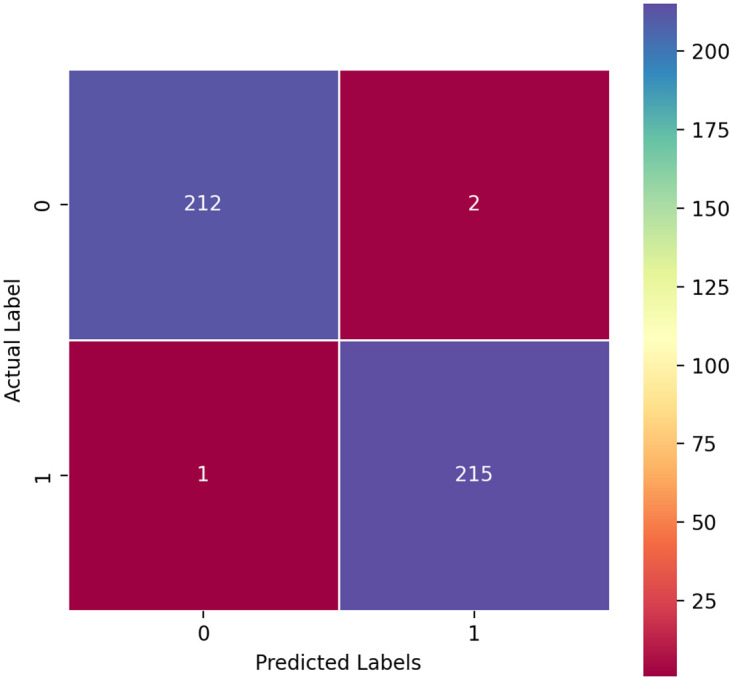
The confusion matrix validation analysis of our purposed model.

The validation of our proposed CART model, as illustrated in the confusion matrix presented in Fig 5, demonstrates the efficacy of our approach in mango orchard detection. The analysis reveals that our proposed methodology achieves significantly low error rates, showcasing its superior performance in accurately identifying mango orchards. Various factors including the diversity of environmental conditions, the composition of tree species, and the characteristics of the landscape may significantly impact the efficacy of the detection method across different regions. Hence, while the utilization of Landsat 8 data shows potential for mango tree detection it is imperative to exercise caution and conduct thorough validation across diverse settings to ensure its reliability and suitability for broader applications. Mangoes tree age cannot affect the classification Results. This achievement is particularly noteworthy considering the challenges associated with distinguishing mango orchards from other types of vegetation in satellite images which include variations in canopy structure overlapping spectral signatures, and the influence of seasonal changes on appearance.

### Kfold validations analysis

**Fig 6:**
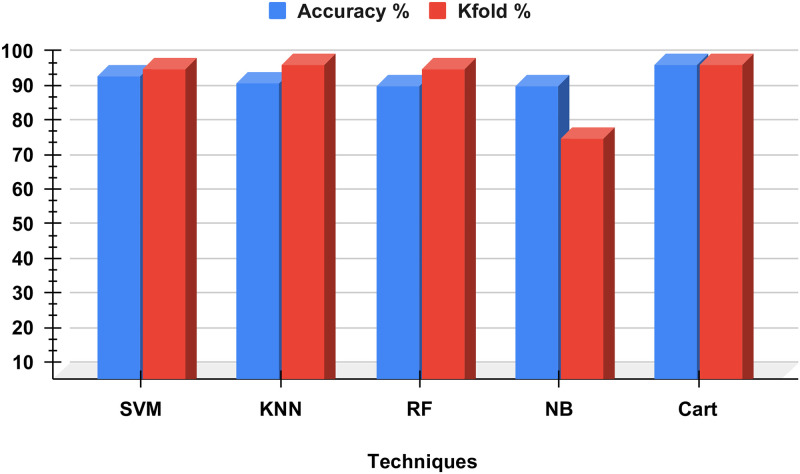
Our study shows K-fold validation analysis.

Table 4 and Fig 6 show the Kfold Validation Analysis. To assess whether or not the machine learning models are employed. We used a technique called k-fold cross-validation that has been examined in the past. The validation is carried out using each of the dataset’s five folds.

**Table 4:** Our study shows K-Fold validation.

K-Fold	Technique	Accuracy	K-Fold Accuracy
5	SVM	96	98
5	RF	99	99
5	KN	94	98
5	NB	93	78
5	Cart	99	99

The visualization of the results of the analysis reveals that the SVM model achieved an accuracy of 96% and that it is possible to get an accuracy of 98 Percent using k-fold 99% using Cart results and 100 Percent using Random forest. Throughout the entirety of this investigation the Naive Bayes models obtained the worst possible accuracy scores. In conclusion, the k-fold procedure is implemented to validate every one of the models that are utilized. According to the findings of the k-fold investigation, the machine learning models we are utilizing do not have any issues with overfitting. Models have been generalised to their full potential to offer accurate results based on data that has not been observed. Our study shows accuracy with the professional approach and mechanisms.

### Time complexity analysis

The Cart algorithm uses collaborative learning to provide precise and reliable predictions with minimum time as shown in Table 5. The Cart model stands out for its minimal computing cost compared to other complicated algorithms despite its great predictive performance. Parallelization allows for simultaneous training of many decision trees, increasing efficiency. The approach requires less hyperparameter tuning, leading to a more efficient computational process. Cart’s blend of precision and computational efficiency makes it an ideal answer for machine learning jobs requiring limited resources.

**Table 5:** Our study shows time complexity.

Technique	Training Time (Seconds)
*SVM*	0.039
*KNN*	0.041
*RF*	0.039
*NB*	0.042
**Cart**	**0.036**

### Statistical T-test analysis based validation

To validate the performance results of applied methods against the proposed approach, we performed a statistical T-test-based validation analysis. The results of the analysis are shown in Table 6. The analysis shows that our proposed approach achieved significant performance results compared to the applied KNN and RF methods. The analysis infers that the rejection of the null hypothesis in each instance for mango orchard detection validates the performance results.

**Table 6:** Statistical T-test analysis based validation analysis.

Proposed vs. Others	T-statistic	P-value	Null Hypothesis (*H*_*o*_)
CART vs. KNN	16.80	0.003	Rejected
CART vs. RF	14.50	0.005	Rejected

### Comparative analysis with state-of-the-art

Table 7 examines the comparative state-of-the-art research that is applied. The comparative parameters are the learning type purposed technique, accuracy, recall and F1 score. The data shows that, as compared to earlier strategies, our targeted strategy the outperformed Cart model achieved the greatest score. Our intended model outperformed cutting-edge research. The confusion matrix analysis is carried out to verify our performance matrix score as shown in the analysed figure.

**Table 7:** Comparisons based on state-of-the-art approaches performance.

Ref	Year	Satellite	Proposed Technique	Performance Score
[7]	2020	Landsat-8	Principal component Analysis	92%
[11]	2020	Landsat-8	Support vector Machine	92%
[12]	2023	Landsat-8	Convolutional Neural Network	84%
[14]	2022	Landsat-8	Principal component Analysis	95%
[15]	2022	Sentinel-1, 2	Random Forest	91%
**Our**	2024	**Landsat-8**	**Cart**	**99%**

### Area estimation and mapping

**Fig 7:**
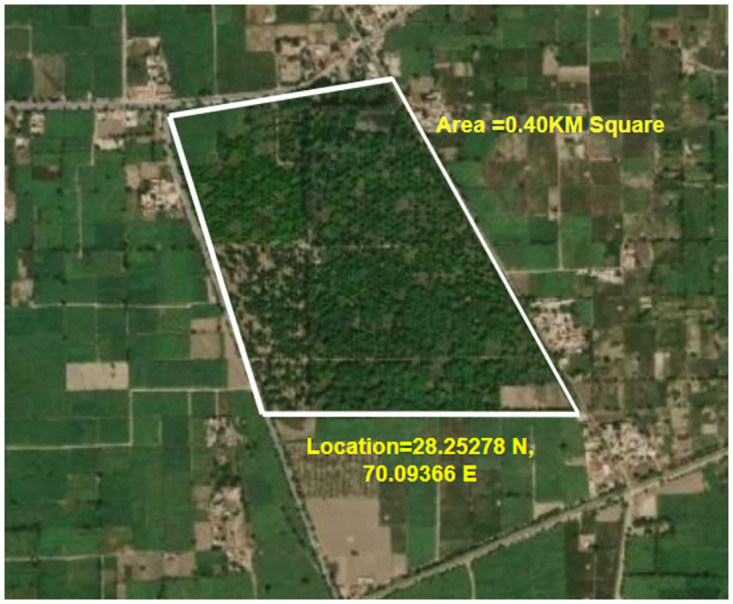
Our study shows the calculated area estimation.

Each class can easily estimate the number of pixels covered by a categorized image as shown in Fig 7. The following formula can be used to calculate the area of a certain class. Ac equals Pc*r*r/100000. Where Ac is the entire land area of class C in hectares, Pc is the total number of pixels in class C and r is the image resolution. These two values represent the image’s overall quality. Our approximate area is 0.40 km2. We convert the estimated area to 0.4 hectare = 0.004 km^2^.

**Fig 8:**
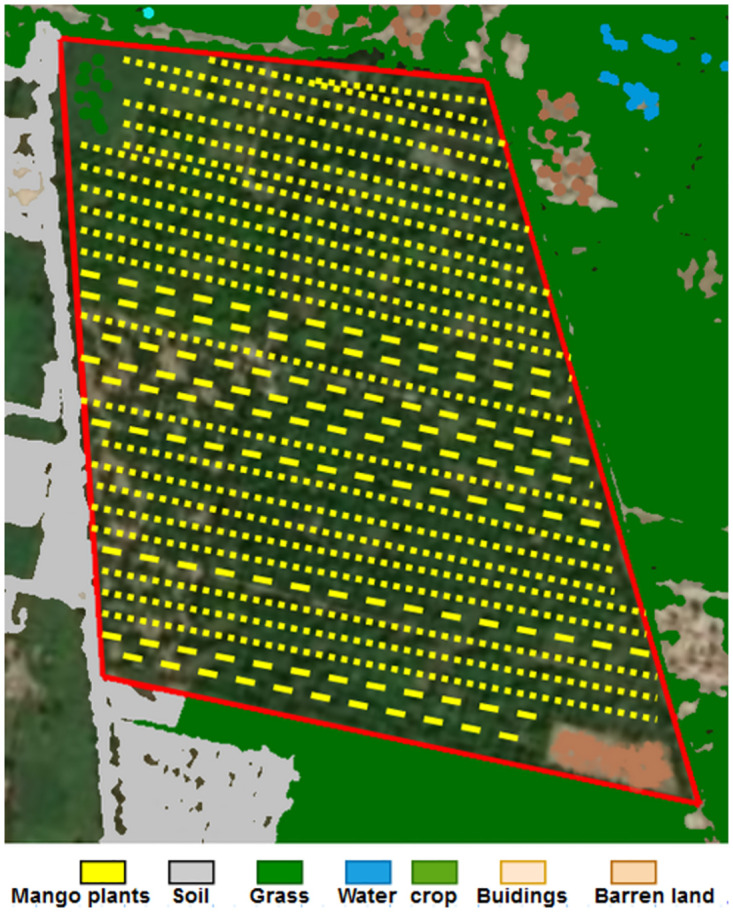
Our study shows classification area map results.

In addition, we have provided mapping results that depict the spatial pattern of classified mango trees, illustrating areas of accuracy and potential errors. The map results are illustrated in Fig 8. In the case of applying the proposed method to other areas with similar environments, it’s Easy to assess its adaptability and generalizability. Factors such as variations in tree species, canopy density, and environmental conditions could not influence the method’s performance. Regarding the age of mango trees, it’s reasonable to expect that age could not indeed impact classification results. Therefore, accounting for tree age variability could not be essential for achieving accurate and reliable classification results across different age cohorts of mango trees.

## Conclusion and future work

This research uses machine learning to predict the identification of Mango plants based on data collected from 2150 Mango tree samples using Landsat 8. The five Machine Learning models namely Random Forest, K-Nearest Neighbor, Cart, Naïve Byes and Support Vector Machine are applied and compared. The proposed Cart algorithm achieves a processing time of 0.036 seconds with 99% accuracy by utilizing a purpose-built feature selection technique. The suggested model surpasses the state-of-the-art studies when compared. The proposed model is being evaluated using the 5-fold cross-validation technique to assess overfitting. The results imply that remotely sensed data can be utilized for plant detection. The research aids the food and agriculture sector in resource management.

### Future work

The study’s limitations and our plans to enhance the dataset involve gathering supplementary data on mango trees and employing data balancing techniques. Furthermore, the deep learning-based approach will be employed for plant prediction.

## Supporting information

S1 Data Mango orchard dataset Landsat-8. (CSV)
